# Case report: Rapid clinical improvement of anti-HMGCR immune-mediated necrotizing myopathy treated with efgartigimod

**DOI:** 10.3389/fimmu.2024.1495415

**Published:** 2024-11-06

**Authors:** Quantao Zeng, Kai Chen, Li Zeng, Lixia Xu, Song Tan

**Affiliations:** ^1^ Department of Neurology, Sichuan Provincial People’s Hospital, University of Electronic Science and Technology of China, Chengdu, China; ^2^ Rare Disease Medical Center, Sichuan Provincial People’s Hospital, University of Electronic Science and Technology of China, Chengdu, China

**Keywords:** HMGCR, IMNM, efgartigimod, exacerbation, IIM

## Abstract

Immune-mediated necrotizing myopathy (IMNM) with anti-HMGCR antibody positivity is characterized by proximal extremity weakness, increased creatine kinase, and extensive muscle edema. There is an urgent need to find more appropriate treatment options for anti-HMGCR IMNM patients who do not respond well to conventional therapy in the acute phase. With the advent of targeted biologics, new treatment options are available. We report on a 66-year-old anti-HMGCR IMNM patient who initially presented with a 1-month history of progressive proximal extremity weakness and dysphagia with markedly elevated creatine kinase. The patient did not respond to conventional high-dose glucocorticoid and intravenous immunoglobulin therapy, and his symptoms rapidly deteriorated over the 2 weeks after this treatment, with worsening limb weakness that prevented walking, marked proximal muscle atrophy, and weight loss. After one cycle (four infusions) of efgartigimod, the patient’s symptoms improved markedly and he has since (for several months) remained in a good clinical state.

## 1 Introduction

Idiopathic inflammatory myopathies (IIMs) are a rare group of autoimmune diseases that primary affect the muscles but can also affect multiple organs such as the skin, joints, lungs, gastrointestinal tract, and heart. The muscle manifestations can cause severe impairment, while the extra-muscular manifestations can lead to life-threatening complications (1). Initially, IIMs were classified as dermatomyositis or polymyositis (2). However, with the identification of anti-signal recognition particle (anti-SRP) antibodies (3), another subgroup, designated immune-mediated necrotizing myopathies (IMNM) (4), became independent from polymyositis.

IMNM primarily involves muscle fiber necrosis, with minimal lymphocytic infiltration (5). Later, another myositis-specific antibody, anti-3-hydroxy-3-methylglutaryl-CoA reductase (anti-HMGCR), was also found to be characteristic of IMNM (it was first identified in 38 patients with pathologic features of IMNM) (6). IMNM is now categorized into three subgroups based on serum antibody levels: anti-SRP antibody-positive, anti-HMGCR antibody-positive, and antibody-negative IMNM.

With the advent of an increasing number of targeted biologics, the treatment of immune-based diseases (particularly those with well-defined pathogenic antibodies) has entered a new era. Efgartigimod was the first approved and marketed neonatal Fc receptor (FcRn) antagonist. Its mechanism of action is to accelerate the degradation of IgG, thereby reducing the levels of disease-causing antibodies. It is approved and marketed in several countries, and in most of them, its indication is generalized myasthenia gravis (gMG) with detectable anti-acetylcholine receptor (AChR) antibody (7).

The lack of clinical trial evidence on anti-HMGCR IMNM treatment means that evidence is typically obtained from case series, cohort studies, and expert opinion (8). Owing to the presence of the pathogenic anti-HMGCR IgG antibody (9), efgartigimod has strong therapeutic potential for anti-HMGCR IMNM, especially in patients with acute exacerbation and poor responses to glucocorticoid and intravenous immunoglobulin (IVIG) treatment. There are no previous studies or case reports on the use of efgartigimod in patients with anti-HMGCR IMNM.

Here, we report on a case of a 66-year-old male patient with anti-HMGCR IMNM. He had a 1-month history of progressive proximal extremity weakness and pharyngeal muscle involvement manifesting as difficulty swallowing. He did not respond to high-dose glucocorticoid and IVIG treatment, and his symptoms rapidly progressed over the 2 weeks after this treatment. However, after one cycle (four infusions) of efgartigimod, his symptoms were effectively resolved.

## 2 Case description

A 66-year-old man presented in a wheelchair with a 1-month history of progressive weakness and dysphagia (Jan 30, 2024). The weakness started in his left lower limb. Days later, he was unable to lift his arm above his shoulder. He then had some difficulty walking and, later, reported some difficulty swallowing. He reported no recent illness or travel, toxin exposure, insect bites, tobacco/alcohol addiction, or family history of similar presentations. No recent weight loss was noted. He was taking oral valsartan to control hypertension and atorvastatin (10 mg/d) to control hyperlipidemia. Surgical history included appendicectomy and cholecystectomy.

Neurological examination revealed age-appropriate mental status. He had some difficulty swallowing (Water Swallow Test: grade 2). He had symmetrical weakness in all four limbs, with proximal muscle strength of grade 4- and distal muscle strength of grade 5- (Manual Muscle Testing-8 [MMT8]: 63 out of 80). All other physical examinations were normal.

**Figure 1:**
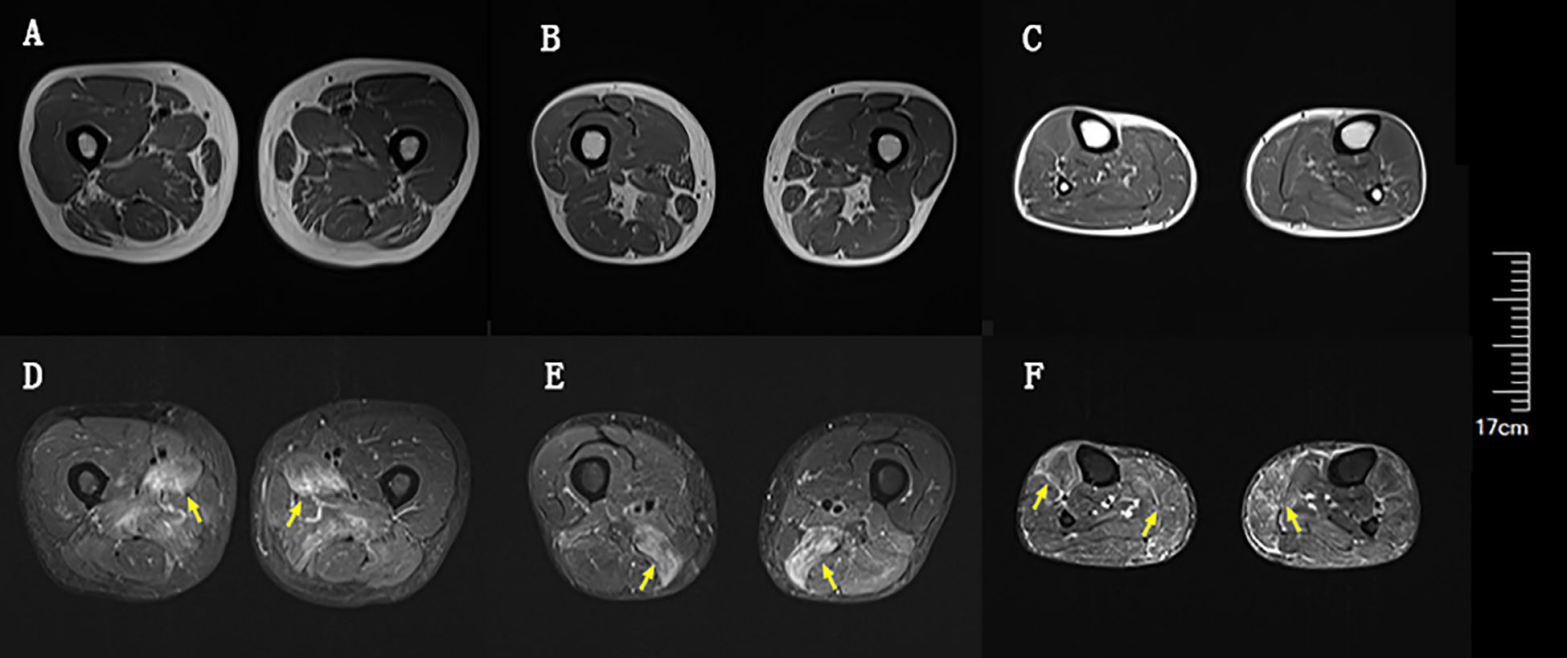
Diffuse muscle edema of thighs and calves on magnetic resonance imaging (MRI) using short tau inversion recovery (STIR). **(A)** Upper portion of thighs on T1. **(B)** Lower portion of thighs on T1. **(C)** Calves on T1. **(D)** Upper portion of thighs on STIR. **(E)** Lower portion of thighs on STIR. **(F)** Calves on STIR. Yellow arrows indicate muscle edema.

**Figure 2:**
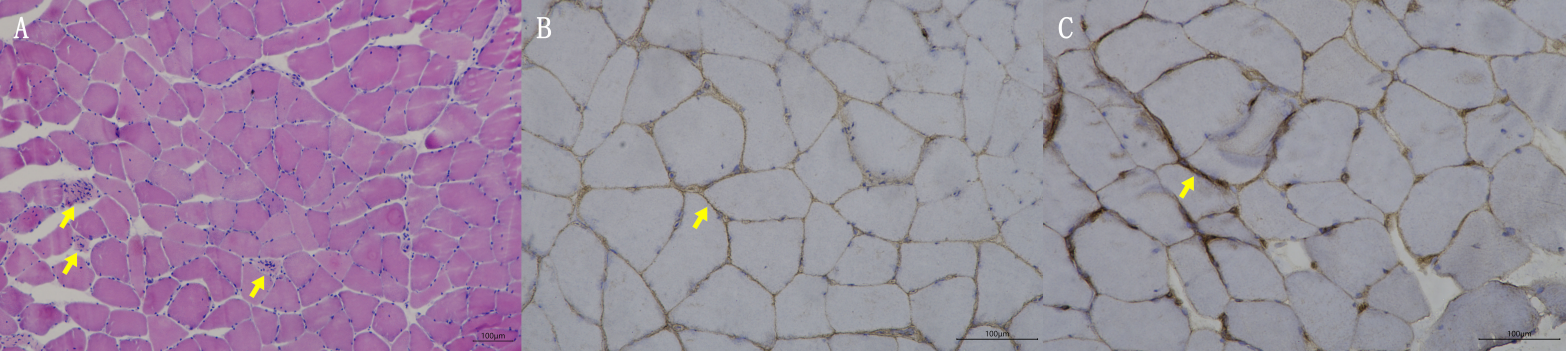
Muscle biopsy pathologic findings. **(A)** HE staining; yellow arrows indicate muscle fiber necrosis. **(B)** Membrane attack complex staining; yellow arrow indicates deposition of membrane attack complex. **(C)** Major histocompatibility complex I staining; yellow arrow indicates expression of major histocompatibility complex I.

Blood tests at admission were remarkable, with creatine kinase (CK) of 6859 U/L (reference range: 50–310), creatine kinase isoenzyme MB (CK-MB) of 250.1 U/L (reference range: 0–25), troponin-T of 508 ng/L (reference range: 0–16.8), myoglobin of 2802 ng/mL (reference range: <72), alanine aminotransferase of 336 U/L (reference range: 9–50), aspartic transaminase of 289 U/L (reference range: 15–40), erythrocyte sedimentation rate of 40 mm/h (reference range: 0–15), and IgG of 36.8 g/L (reference range: 7–16). Other tests, including complete blood count, kidney function, electrolytes, coagulation profile, blood glucose, hemoglobin A1c, thyroid function tests, infectious disease screening, immunofixation electrophoresis, anti-neutrophil cytoplasmic antibody, autoimmune hepatitis antibody profile, and urine analysis, were normal. Electrocardiography and echocardiography showed no abnormalities. Electromyography was normal. Muscle magnetic resonance imaging (MRI) using short tau inversion recovery (STIR) showed diffuse, patchy areas with slightly longer T2 signals in the posterior portions of the thighs and calves (**Figure 1**). We then performed a muscle biopsy on the left gastrocnemius muscle. Muscle pathology suggested muscle fiber necrosis (**Figure 2**). Finally, serological immune testing (involving an enzyme-linked immunosorbent assay) indicated an anti-HMGCR antibody level of 657.8 U/ml (reference value: <185 U/mL). The final diagnosis was anti-HMGCR IMNM.

First, atorvastatin was discontinued. Next, to relieve the patient’s symptoms, we immediately started high-dose glucocorticoid treatment (methylprednisolone 1000 mg for 3 d, followed by 500 mg for 2 d) and IVIG (2 g/kg over 5 d). Unexpectedly, the patient’s symptoms did not get better after starting these treatments, though they did not worsen during the treatment. There were slight decreases in CK (1051 U/L), CK-MB (98 U/L), troponin-T (239 ng/L), myoglobin (1037 ng/mL), and aspartic transaminase (189 U/L). The patient was discharged (Feb 8) with oral prednisone (60 mg/d) and mycophenolate mofetil (0.5 g twice/d) for the start of the traditional Chinese New Year festival. However, in the 2 weeks after discharge, he rapidly developed limb weakness, neck muscle weakness, and difficulty standing and swallowing (MMT8: 48 out of 80; Water Swallow Test: grade 4). He had some proximal muscle atrophy in the limbs and weight loss of 7 kg due to reduced eating caused by dysphagia.

After comprehensive consideration, we initiated efgartigimod (Feb 23) to provide rapid relief, given the deterioration of the patient’s condition and the limited effectiveness of conventional therapy. The patient completed one cycle (four infusions) of efgartigimod (10 mg/kg, 75 kg, once a week for 4 weeks). Unexpectedly, the patient’s dysphagia was relieved 3 d after starting efgartigimod. The patient’s limb weakness also progressively improved throughout the period in which the four efgartigimod infusions (which were on Feb 23, Mar 1, Mar 8, and Mar 15) were administered. The IgG level decreased from 16.8 g/L just before the first efgartigimod infusion to 3.42 g/L after the last efgartigimod infusion.

**Figure 3:**
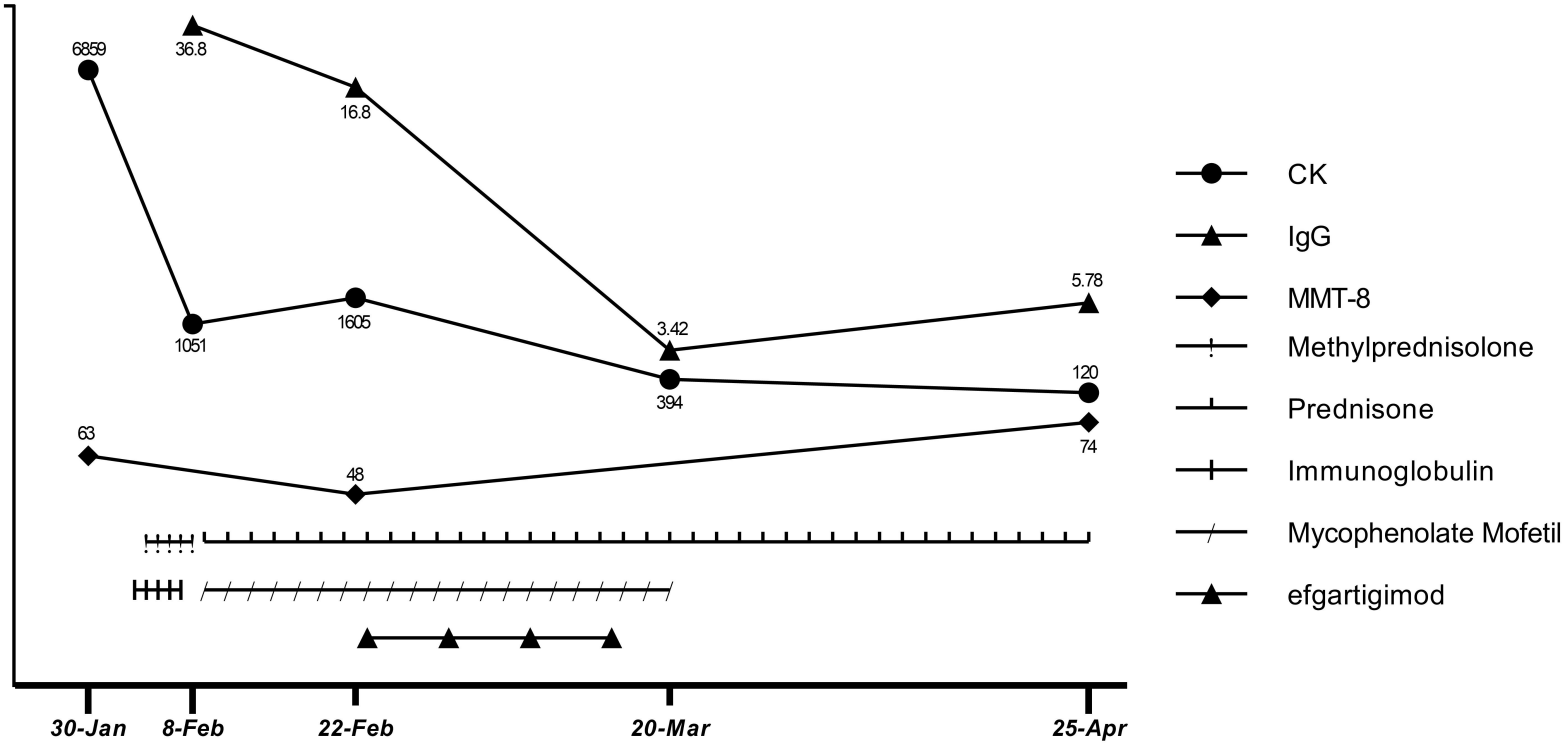
Therapeutic process and changes in CK, IgG levels, and MMT-8 score.

As his employer provided him with a free medical checkup, he was admitted to the Rheumatology and Immunology Department of another hospital in late March. The doctor recommended that he stop taking mycophenolate mofetil. As his symptoms and biochemical indicators continued to improve and he remained in a good clinical condition, we did not ask him to restart mycophenolate mofetil. At a follow-up appointment on Apr 25, he had almost completely recovered (MMT8: 74 out of 80, Water Swallow Test: grade 1). He had no difficulty walking and had regained his pre-illness weight. His hematological indicators had almost normalized, with CK of 120 U/L, CK-MB of 12.9 U/L, troponin-T of 231 ng/L, myoglobin of 102 ng/mL, aspartic transaminase of 17 U/L, alanine aminotransferase of 21 U/L, and IgG of 5.78 g/L (Apr 25). The detailed treatment course and index changes are shown below (**Figure 3**). As of Jun 17, he was only taking low-dose prednisone (10 mg/d) and was scheduled to taper the prednisone.

## 3 Discussion

Anti-HMGCR IMNM is rare. A recent study reported an incidence ranging from 0.9 to 2.4 per million per year (10). Among patients with IIM, the prevalence of anti-HMGCR IMNM ranges from 6% to 10% (6, 11, 12). The vast majority of patients with anti-HMGCR IMNM exhibit proximal extremity weakness and elevated CK (6). Dysphagia occurs in up to a quarter of these patients (11, 13). Although cardiac and skin involvement can occur (11, 14), most cohorts of these patients have a very low rate of extra-muscular manifestations (15–17). Therefore, extra-muscular involvement is not currently considered to be a key part of the disease spectrum of anti-HMGCR IMNM. Two studies suggested that there was only a mildly increased risk of malignancy in patients with anti-HMGCR IMNM (17, 18). Current consensus guidelines categorize anti-HMGCR autoantibody as an autoantibody that poses intermediate risk regarding malignancy, and they recommend basic and intensive screening at diagnosis (19). Usually, widespread muscle edema in the thighs with involvement of the anterior compartment can be seen on MRI (20). Our patient’s muscle involvement was primarily in the posterior portion of the thighs and calves.

As statins and anti-HMGCR antibody share the same target, statins may be a trigger underlying the development of anti-HMGCR IMNM. The rate of statin exposure among anti-HMGCR IMNM patient cohorts ranges from 15% to 65% depending on their geographic origin (6, 21). Adult anti-HMGCR IMNM patient cohorts in Asia generally have lower rates of statin exposure (18, 21–24). Our patient was a relatively typical anti-HMGCR IMNM patient, presenting with a history of statin exposure, proximal extremity weakness, high CK levels, no extra-muscular manifestations, no associated tumors, and extensive edema of the legs on MRI.

There is a lack of clinical trial evidence on effective treatment for anti-HMGCR IMNM, so evidence is usually derived from case series, cohort studies, and expert opinion. The consensus guidelines on anti-HMGCR IMNM treatment reported at the 224th European Neuromuscular Centre (ENMC) International Workshop in 2016 recommend initial oral or intravenous glucocorticoid therapy for severe disease (8), with the initiation of immunosuppressant treatment (such as methotrexate, azathioprine, or mycophenolate mofetil) concurrently or within one month (8). As some observational studies found that steroids failed to control disease in the vast majority (92–100%) of patients (25–27), additional IVIg can also be considered. If the response after 6 months is inadequate, rituximab can be added (8). After achieving disease control, it is recommended that the glucocorticoid treatment be tapered to the minimum dose that remains effective and an immunosuppressant or rituximab should be continued until at least 2 years of well-controlled disease has been achieved (8).

Considering the patient’s high anti-HMGCR antibody level, we combined high-dose intravenous glucocorticoid and IVIG (both for 5 d) as the initial treatment, followed by oral prednisone and mycophenolate mofetil. We were expecting a good outcome but, surprisingly, the patient’s symptoms rapidly worsened over the 2 weeks after the initial glucocorticoid and IVIG treatment. According to the consensus guidelines (8), rituximab should have been started. However, based on previous experience of its pharmacologic effects, rituximab was unlikely to provide rapid relief. In seropositive IMNM, the antibody titer correlates with disease activity (28). The anti-HMGCR antibody titer correlates with muscle strength and CK levels (11, 13). *In vitro* and vivo experiments have also shown that anti-HMGCR autoantibody is pathogenic (9, 29). Moreover, muscle fiber necrosis in IMNM is an antibody- and complement-dependent process (30). It has been shown that efgartigimod can restores muscle function (31). Therefore, it is reasonable to hypothesize that lowering the serum anti-HMGCR antibody level could contribute to rapid disease control. Plasma exchange and efgartigimod are two ways to rapidly lower the serum antibody level. Given the invasive nature of plasma exchange, the patient selected efgartigimod. Fortunately, the patient’s symptoms resolved rapidly and he was glad to have chosen efgartigimod.

Efgartigimod is an FcRn antagonist that competitively binds to FcRn. FcRn is a multifunctional Fc-γ receptor that binds to circulating IgG antibodies, reduces their degradation in lysosomes, and releases them into the extracellular space, thereby prolonging their half-life (32). Thus, inhibition of FcRn can increase the catabolism of IgG (including pathogenic autoantibodies), providing targeted therapy for immune-mediated diseases, especially those with well-defined pathogenic antibodies (33). The efficacy of efgartigimod has been demonstrated in a series of clinical trials (34–36). In particular, the ADAPT study (34) demonstrated the favorable effects of efgartigimod in gMG, laying the foundation for its marketing approval for treating gMG with detectable anti-AChR antibody. Efgartigimod is highly effective at reducing patients’ IgG levels, as evidenced by the large decrease in IgG levels in our patient from 16.8 to 3.42 g/L. Besides gMG, efgartigimod has been used to treat neuromyelitis optica spectrum disorder, Guillain–Barré syndrome, chronic inflammatory demyelinating polyneuropathy, and stiff-person syndrome (37–40). However, there was no precedent for the use of efgartigimod in individuals with IMNM, though efgartigimod restored muscle function in a humanized mouse model of IMNM (31). This is the first case report on the use of efgartigimod in an anti-HMGCR IMNM patient. The patient’s favorable response to efgartigimod provides new insights into a potentially effective treatment for anti-HMGCR IMNM patients with poor responses to traditional treatments.

Treatment options for anti-HMGCR IMNM are limited. When glucocorticoid and IVIG treatment are not effective at preventing exacerbation, using efgartigimod to rapidly reduce pathogenic antibody levels for symptomatic relief appears to be a better option than rituximab. After the last efgartigimod infusion (Mar 15), there was a gradual increase in IgG levels (from 3.42 to 5.78 g/L) over 36 d (from Mar 15 to Apr 25). At the follow-up appointment on Jun 17, which was 3 months after the last efgartigimod infusion, the patient remained in good clinical condition. We hypothesize that despite IgG levels increasing, the anti-HMGCR autoantibody level had not yet highly increased. Notably, the effects of the prior use of glucocorticoid and IVIG treatment should not be ignored when considering the patient’s clinical improvement. Glucocorticoid treatment helps to prevent the immune system from producing further pathogenic antibodies, and IVIG reduces myofiber necrosis caused by complement. Thus, in this particular context, an immediate effect could be achieved by using efgartigimod to quickly remove circulating pathogenic antibodies from the blood. Therefore, we cannot attribute the patient’s recovery solely to the effects of efgartigimod. It is worth noting that efgartigimod treatment was initiated only half a month after IVIG administration. We know that efgartigimod is potent in reducing IgG levels, so efgartigimod certainly has some effects on the administered IVIG. However, because IVIG did not achieve the expected therapeutic effect, we do not think this is a critical factor in the treatment. The patient discontinued mycophenolate mofetil in late March because rheumatologists were prepared to switch him from mycophenolate mofetil to cyclophosphamide and immunoglobulin, but as he experienced sustained symptom remission after withdrawal of mycophenolate mofetil, cyclophosphamide and immunoglobulin were not administered. As of Jun 17, the patient remained in a good clinical condition while taking low-dose prednisone (10 mg/d) and was on a schedule to taper the prednisone until withdrawal.

Although no significant side effects were observed in our patient, the ADAPT study showed that upper respiratory tract and urinary tract infections were common side effects of efgartigimod (32). Because efgartigimod can significantly reduce the level of IgG, the CD4-positive T cell level should be checked during the treatment of efgartigimod, and prophylactic anti-infective treatment may be used if necessary.

## 4 Conclusion

There are few cases of anti-HMGCR IMNM, which contributes to the lack of an evidence base for the standardized treatment of this disease. The choice of treatment is even more tricky for patients who do not respond well to conventional treatment. As a new targeted biologic, efgartigimod has shown strong efficacy for treating gMG and has led to promising results in many immune-mediated disorders (especially those involving well-defined pathogenic IgG antibody). There are no previous studies or case reports on the use of efgartigimod in patients with anti-HMGCR IMNM, and our case provides some limited experience in this regard. However, the optimum management of acute deterioration in anti-HMGCR IMNM patients and the use of long-term maintenance therapy for anti-HMGCR IMNM still needs further studies.

## Data Availability

The raw data supporting the conclusions of this article will be made available by the authors, without undue reservation.

## References

[1] Dobloug C Garen T Bitter H Stjärne J Stenseth G Grøvle L. Prevalence and clinical characteristics of adult polymyositis and dermatomyositis; data from a large and unselected Norwegian cohort. Ann Rheum Dis. (2015) 74:1551–6. doi: 10.1136/annrheumdis-2013-205127 24695011

[2] Bohan A Peter JB. Polymyositis and dermatomyositis (first of two parts). N Engl J Med. (1975) 292:344–7. doi: 10.1056/NEJM197502132920706 1090839

[3] Reeves WH Nigam SK Blobel G. Human autoantibodies reactive with the signal-recognition particle. Proc Natl Acad Sci U.S.A. (1986) 83:9507–11. doi: 10.1073/pnas.83.24.9507 PMC387169 2432596

[4] Hoogendijk JE Amato AA Lecky BR Choy EH Lundberg IE Rose MR. 119th ENMC international workshop: trial design in adult idiopathic inflammatory myopathies, with the exception of inclusion body myositis, 10-12 october 2003, Naarden, the Netherlands. Neuromuscul Disord. (2004) 14:337–45. doi: 10.1016/j.nmd.2004.02.006 15099594

[5] Miller T Al-Lozi MT Lopate G Pestronk A. Myopathy with antibodies to the signal recognition particle: clinical and pathological features. J Neurol Neurosurg Psychiatry. (2002) 73:420–8. doi: 10.1136/jnnp.73.4.420 PMC1738058 12235311

[6] Mammen AL Chung T Christopher-Stine L Rosen P Rosen A Doering KR. Autoantibodies against 3-hydroxy-3-methylglutaryl-coenzyme A reductase in patients with statin-associated autoimmune myopathy. Arthritis Rheum. (2011) 63:713–21. doi: 10.1002/art.30156 PMC3335400 21360500

[7] Heo Y-A. Efgartigimod alfa in generalised myasthenia gravis: a profile of its use. CNS Drugs. (2023) 37:467–73. doi: 10.1007/s40263-023-01000-z PMC10212825 37000339

[8] Allenbach Y Mammen AL Benveniste O Stenzel W Immune-Mediated Necrotizing Myopathies Working Group. ENMC international workshop. 224th ENMC international workshop: clinico-sero-pathological classification of immune-mediated necrotizing myopathies Zandvoort, the Netherlands, 14-16 october 2016. Neuromuscul Disord. (2018) 28:87–99. doi: 10.1016/j.nmd.2017.09.016 29221629

[9] Bergua C Chiavelli H Allenbach Y Arouche-Delaperche L Arnoult C Bourdenet G. *In vivo* pathogenicity of IgG from patients with anti-SRP or anti-HMGCR autoantibodies in immune-mediated necrotising myopathy. Ann Rheum Dis. (2019) 78:131–9. doi: 10.1136/annrheumdis-2018-213518 30309969

[10] Khoo T Chinoy H. Anti-HMGCR immune-mediated necrotising myopathy: addressing the remaining issues. Autoimmun Rev. (2023) 22:103468. doi: 10.1016/j.autrev.2023.103468 37884200

[11] Allenbach Y Drouot L Rigolet A Charuel JL Jouen F Romero NB. Anti-HMGCR autoantibodies in European patients with autoimmune necrotizing myopathies: inconstant exposure to statin. Medicine. (2014) 93:150–7. doi: 10.1097/MD.0000000000000028 PMC4632910 24797170

[12] Watanabe Y Uruha A Suzuki S Nakahara J Hamanaka K Takayama K. Clinical features and prognosis in anti-SRP and anti-HMGCR necrotising myopathy. J Neurol Neurosurg Psychiatry. (2016) 87:1038–44. doi: 10.1136/jnnp-2016-313166 27147697

[13] Tiniakou E Pinal-Fernandez I Lloyd TE Albayda J Paik J Werner JL. More severe disease and slower recovery in younger patients with anti-3-hydroxy-3-methylglutaryl-coenzyme a reductase-associated autoimmune myopathy. Rheumatol (Oxf Engl). (2017) 56:787–94. doi: 10.1093/rheumatology/kew470 PMC5850825 28096458

[14] Szczesny P Barsotti S Nennesmo I Danielsson O Dastmalchi M. Screening for anti-HMGCR antibodies in a large single myositis center reveals infrequent exposure to statins and diversiform presentation of the disease. Front Immunol. (2022) 13:866701. doi: 10.3389/fimmu.2022.866701 35603214 PMC9114810

[15] Aggarwal R Moghadam-Kia S Lacomis D Malik A Qi Z Koontz D. Anti-hydroxy-3-methylglutaryl-coenzyme a reductase (anti-HMGCR) antibody in necrotizing myopathy: treatment outcomes, cancer risk, and role of autoantibody level. Scand J Rheumatol. (2020) 49:405–11. doi: 10.1080/03009742.2019.1672782 31801390

[16] Gupta L Nune A Naveen R Verma R Prasad P Kharbanda R. The prevalence and clinical characteristics of anti-HMGCR (anti-3-hydroxy-3-methyl-glutaryl-coenzyme a reductase) antibodies in idiopathic inflammatory myopathy: an analysis from the myocite registry. Rheumatol Int. (2022) 42:1143–54. doi: 10.1007/s00296-021-05063-3 35031847

[17] Allenbach Y Keraen J Bouvier A-M Jooste V Champtiaux N Hervier B. High risk of cancer in autoimmune necrotizing myopathies: usefulness of myositis specific antibody. Brain. (2016) 139:2131–5. doi: 10.1093/brain/aww054 27086869

[18] Kadoya M Hida A Hashimoto Maeda M Taira K Ikenaga C Uchio N. Cancer association as a risk factor for anti-HMGCR antibody-positive myopathy. Neurol Neuroimmunol Neuroinflamm. (2016) 3:e290. doi: 10.1212/NXI.0000000000000290 27761483 PMC5056647

[19] Oldroyd AGS Callen JP Chinoy H Chung L Fiorentino D Gordon P. International guideline for idiopathic inflammatory myopathy-associated cancer screening: an international myositis assessment and clinical studies group (imacs) initiative. Nat Rev Rheumatol. (2023) 19:805–17. doi: 10.1038/s41584-023-01045-w PMC10834225 37945774

[20] Lee K-H Gao Y Lau V. Statin-associated anti-3-hydroxy-3-methyl-glutaryl-coenzyme a reductase (HMGCR) myopathy: imaging findings on thigh-muscle magnetic resonance imaging (MRI) in six patients. Muscle Nerve. (2021) 64:500–4. doi: 10.1002/mus.27382 34297419

[21] Ge Y Lu X Peng Q Shu X Wang G. Clinical characteristics of anti-3-hydroxy-3-methylglutaryl coenzyme A reductase antibodies in Chinese patients with idiopathic inflammatory myopathies. PloS One. (2015) 10:e0141616. doi: 10.1371/journal.pone.0141616 26509687 PMC4624805

[22] Liang W-C Wang C-H Chen W-Z Kuo YT Lin HF Suzuki S. Treatment experience of Taiwanese patients with anti-3-hydroxy-3-methylglutaryl-coenzyme a reductase myopathy. Kaohsiung J Med Sci. (2020) 36:649–55. doi: 10.1002/kjm2.12240 PMC11896125 32666706

[23] Watanabe Y Suzuki S Nishimura H Murata K-Y Kurashige T Ikawa M. Statins and myotoxic effects associated with anti-3-hydroxy-3-methylglutaryl-coenzyme a reductase autoantibodies: an observational study in Japan. Medicine. (2015) 94:e416. doi: 10.1097/MD.0000000000000416 25634171 PMC4602975

[24] Oh EK Lee S-A Lee HJ Cha YJ Kim S Lee H-S. Clinical and radiological features of Korean patients with anti-HMGCR myopathy. J Clin Neurol. (2023) 19:460–8. doi: 10.3988/jcn.2022.0374 PMC10471552 36929062

[25] Allenbach Y Benveniste O. Acquired necrotizing myopathies. Curr Opin Neurol. (2013) 26:554–60. doi: 10.1097/WCO.0b013e328364e9d9 23995277

[26] Grable-Esposito P Katzberg HD Greenberg SA Srinivasan J Katz J Amato AA. Immune-mediated necrotizing myopathy associated with statins. Muscle Nerve. (2010) 41:185–90. doi: 10.1002/mus.21486 19813188

[27] Ramanathan S Langguth D Hardy TA Garg N Bundell C Rojana-Udomsart A. Clinical course and treatment of anti-HMGCR antibody-associated necrotizing autoimmune myopathy. Neurol Neuroimmunol Neuroinflamm. (2015) 2:e96. doi: 10.1212/NXI.0000000000000096 25866831 PMC4386794

[28] Allenbach Y Benveniste O Stenzel W Boyer O. Immune-mediated necrotizing myopathy: clinical features and pathogenesis. Nat Rev Rheumatol. (2020) 16:689–701. doi: 10.1038/s41584-020-00515-9 33093664

[29] Arouche-Delaperche L Allenbach Y Amelin D Preusse C Mouly V Mauhin W. Pathogenic role of anti-signal recognition protein and anti-3-hydroxy-3-methylglutaryl-coa reductase antibodies in necrotizing myopathies: myofiber atrophy and impairment of muscle regeneration in necrotizing autoimmune myopathies. Ann Neurol. (2017) 81:538–48. doi: 10.1002/ana.24902 28224701

[30] Allenbach Y Arouche-Delaperche L Preusse C Radbruch H Butler-Browne G Champtiaux N. Necrosis in anti-SRP(+) and anti-HMGCR(+)myopathies: role of autoantibodies and complement. Neurology. (2018) 90:e507–17. doi: 10.1212/WNL.0000000000004923 29330311

[31] Julien S Woning B De Ceuninck L Briand E Jaworski T Roussel G. Efgartigimod restores muscle function in a humanized mouse model of immune-mediated necrotizing myopathy. Rheumatol (Oxford). (2023) 62:4006–11. doi: 10.1093/rheumatology/kead298 37335864

[32] Roopenian DC Akilesh S. FcRn: the neonatal Fc receptor comes of age. Nat Rev Immunol. (2007) 7:715–25. doi: 10.1038/nri2155 17703228

[33] Zuercher AW Spirig R Baz Morelli A Rowe T Käsermann F. Next-generation fc receptor-targeting biologics for autoimmune diseases. Autoimmun Rev. (2019) 18:102366. doi: 10.1016/j.autrev.2019.102366 31404703

[34] Howard JF Jr Bril V Vu T Karam C Peric S Margania T. Safety, efficacy, and tolerability of efgartigimod in patients with generalised myasthenia gravis (adapt): a multicentre, randomised, placebo-controlled, phase 3 trial. Lancet Neurol. (2021) 20:526–36. doi: 10.1016/S1474-4422(21)00159-9 34146511

[35] Howard JF Jr Bril V Burns TM Mantegazza R Bilinska M Szczudlik A. Randomized phase 2 study of FcRn antagonist efgartigimod in generalized myasthenia gravis. Neurology. (2019) 92:e2661–73. doi: 10.1212/WNL.0000000000007600 PMC6556100 31118245

[36] Saccà F Barnett C Vu T Peric S Phillips GA Zhao S. Efgartigimod improved health-related quality of life in generalized myasthenia gravis: results from a randomized, double-blind, placebo-controlled, phase 3 study (ADAPT). J Neurol. (2023) 270:2096–105. doi: 10.1007/s00415-022-11517-w PMC10025199 36598575

[37] Li Z Xu Q Huang J Zhu Q Yang X Zhang M. Efgartigimod as rescue treatment in acute phase of neuromyelitis optica spectrum disorder: a case report. Heliyon. (2024) 10:e30421. doi: 10.1016/j.heliyon.2024.e30421 38720715 PMC11076956

[38] Zhang H Ma J Feng Y Ma H Liu D Pang X. Efgartigimod in the treatment of Guillain-Barré syndrome. J Neurol. (2024) 271:3506–11. doi: 10.1007/s00415-024-12321-4 38532142

[39] Alfaidi N Karmastaji S Matic A Bril V. FcRn inhibitor therapies in neurologic diseases. CNS Drugs. (2024) 38:425–41. doi: 10.1007/s40263-024-01090-3 38724842

[40] Di Stefano V Alonge P Rini N Militello M Lupica A Torrente A. Efgartigimod beyond myasthenia gravis: the role of FcRn-targeting therapies in stiff-person syndrome. J Neurol. (2024) 271:254–62. doi: 10.1007/s00415-023-11970-1 PMC10769952 37682316

